# Comparative analysis of calcified soft tissues revealed shared deregulated pathways

**DOI:** 10.3389/fnagi.2023.1131548

**Published:** 2023-06-14

**Authors:** Aminat Guseynovna Ibragimova, Yaroslav Mikhailovich Stanishevskiy, Alexey Mikhaylovich Plakkhin, Alexandr Vladimirovich Zubko, Nidal Akhmedovich Darvish, Anton Karenovich Koassary, Anastasia V. Shindyapina

**Affiliations:** ^1^Peoples’ Friendship University of Russia, Moscow, Russia; ^2^MineGenics LLC, Moscow, Russia; ^3^Bakoulev National Medical Research Center for Cardiovascular Surgery, Russian Federation, Moscow, Russia; ^4^Department of Medicine, Brigham and Women’s Hospital, Harvard Medical School, Boston, MA, United States; ^5^Retro Biosciences Inc., Redwood City, CA, United States

**Keywords:** vascular calcification, coronary artery, aorta, pituitary, artery tibial, cardiovascular diseases, PD-1, PD-L1

## Abstract

**Introduction:**

Calcification of soft tissues is a common age-related pathology that primarily occurs within vascular tissue. The mechanisms underlying pathological calcification in humans and tissue specificity of the process is still poorly understood. Previous studies examined calcified tissues on one to one basis, thus preventing comparison of deregulated pathways across tissues.

**Purpose:**

This study aimed to establish common and tissue-specific changes associated with calcification in aorta, artery tibial, coronary artery and pituitary gland in subjects from the Genotype-Tissue Expression (GTEx) dataset using its RNA sequencing and histological data.

**Methods:**

We used publicly available data from the GTEx database https://gtexportal.org/home/aboutGTEx. All GTEx tissue samples were derived by the GTEx consorcium from deceased donors, with age from 20 to 79, both men and women. GTEx study authorization was obtained *via* next-of-kin consent for the collection and banking of de-identified tissue samples for scientific research. Hematoxylin and eosin (H&E) staining of arteries were manually graded based on the presence of calcification on a scale from zero to four, where zero designates absence of calcification and four designates severe calcification. Samples with fat contamination and mislabeled tissues were excluded, which left 430 aorta, 595 artery tibial, 124 coronary artery, and 283 pituitary samples for downstream gene expression analysis. Transcript levels of protein-coding genes were associated with calcification grade using sex, age bracket and cause of death as covariates, and tested for pathway enrichment using gene set enrichment analysis.

**Results:**

We identified calcification deposits in 28 (6.5%) aortas, 121 (20%), artery tibials, 54 (43%), coronary arteries, and 24 (8%) pituitary glands of GTEx subjects. We observed an age-dependent increase in incidence of calcification in all vascular tissues, but not in pituitary. Subjects with calcification in the artery tibial were significantly more likely to have calcification in the coronary artery (OR = 2.56, *p* = 6.3e-07). Markers of calcification previously established in preclinical and *in vitro* studies, e.g., *BMP2* and *RUNX2*, were deregulated in the calcified tibial and coronary arteries, confirming the relevance of these genes to human pathology. Differentially expressed genes associated with calcification poorly overlapped across tissues suggesting tissue-specific nuances in mechanisms of calcification. Nevertheless, calcified arteries unanimously down-regulated pathways of intracellular transport and up-regulated inflammatory pathways suggesting these as universal targets for pathological calcification. In particular, PD-1 and PD-L1 genes were up-regulated in calcified tissues but not in the blood of the same subjects, suggesting that localized inflammation contributes to pathological calcification.

**Conclusion:**

Pathological calcification is a prevalent disease of aging that shares little changes in expression in individual genes across tissues. However, our analysis suggests that it potentially can be targeted by alleviating local inflammation of soft tissues.

## Introduction

Vascular calcification (VC) is associated with classical age-related diseases such as atherosclerosis and type II diabetes (Lanzer et al., 2014; Pescatore et al., 2019), chronic kidney diseases (CKD) (Düsing et al., 2021; Verma et al., 2021), and chronic inflammatory disease (Verma et al., 2021). This deposition of calcium phosphate crystals in the medial and intimal layers of the arteries increases the odds of adverse cardiovascular events (Kwon et al., 2017; Villa-Bellosta and Egido, 2017; Chen et al., 2021).

Pathological calcification is the deposition of calcium salts and the formation of osteo-like structures in soft tissues. Although it can be found in the area of tumors, in the basal ganglia and cerebral cortex (Fahr syndrome; Saleem et al., 2013), pituitary gland, skin (Li et al., 2014), and lungs (Bendayan et al., 2000; Chuang et al., 2021) the most common form is calcification of the cardiovascular system (CVS) (Covic et al., 2007; Rennenberg et al., 2010; New and Aikawa, 2011; Lamprea-Montealegre and Otto, 2021). Another relatively common form of pathological calcification is pituitary calcification. It is a type of brain calcification, which is usually found in pituitary adenomas, intratumoral hematoma, degenerative adenoma tissue and scattered psammoma bodies between the adenoma cells (Garg et al., 2013), prolactinoma, somatotroph adenoma, thyrotroph adenoma and rarely in gonadotroph and corticotroph adenoma (Kurisaka et al., 1986). Most reports of this condition come from Japan and India (Garg et al., 2013).

To date, two main pathological forms of vascular calcification have been described, although they often coexist in the same clinical conditions (Lanzer et al., 2014). The first type is intimal calcification, associated with the development of atherosclerotic plaque, in particular, with the deposition of lipids and cholesterol crystals under the damaged endothelium (Proudfoot and Shanahan, 2001; Lanzer et al., 2014; Kwon et al., 2017). The second type is media calcification, also known as Mönckeberg’s arteriosclerosis, which is characterized by the deposition of calcium salts in the layers of smooth muscles of blood vessels (Demer and Tintut, 2008; Bardeesi et al., 2017; Pérez-Hernández et al., 2017). Meanwhile VC can be also divided into several types based on their histological characteristics: amorphic, chondromorphic, or osteomorphic in structure, and may be characterized as metastatic or dystrophic (Zhu et al., 2012).

Age is a major risk factor for pathological calcification, for example, prevalence of siphon calcification rises from 6% in children <2 years of age to 100% in 90 to 100 year-olds (Bartstra et al., 2020). Other than age, the risk factors include diabetes mellitus, hypercholesterolemia, and a history of cardiovascular disease in both sexes, excessive alcohol intake, and smoking in men, and hypertension in women (Bos et al., 2012). It is particularly interesting that medial and intimal calcification in large intracranial arteries show differences in risk factors and clinical outcomes.

Intracranial artery calcifications are an important predictor of stroke, mortality in stroke patients (Tábuas-Pereira et al., 2018), transient ischemic attacks, epileptic seizures, and cognitive decline (Bugnicourt et al., 2011; Bos et al., 2012, 2014; Nicolas et al., 2015). VC was also shown to have linear positive correlation with Frailty index, a widely used proxy measure of aging (Lee et al., 2020). Furthermore, VC leads to vascular stiffening and impaired vascular compliance, compromised blood circulation. When VC takes place in the aortic arch it leads to hypertension (Barrett et al., 2018). VC in the brain is believed to be contributing to the risk of stroke, dementia and mild cognitive impairment (Aprahamian et al., 2018). VC of blood vessels of limbs may lead to ischemic ulcers, adverse limb events, and cardiovascular mortality (Kao et al., 2015). VC was also shown to be a risk factor to bone fracture in geriatric population, which is believed to be the result of deranged calcium/phosphate metabolism and decreased bone mineral density, which is integral part of pathogenesis of this disease (Kao et al., 2015). Thus, preventing and treating VC is a valuable strategy to improve survival and quality of life in a growing elderly population.

Conventional insight with respect to VC pathophysiology centers around the up-regulation of Runt-related transcription factor 2 (RUNX2; Cai et al., 2016; Hou et al., 2020; Bundy et al., 2021; Chen et al., 2021), a master upstream transcription factor of osteoblasts, and bone morphogenetic protein (BMP) in VSMCs and susceptible cells (Li et al., 2008; Yang et al., 2020). They are exacerbated by persistent inflammation, oxidative stress from advanced glycation or uremic toxin, defective osteoid resorption, and down-regulation of anti-calcific proteins or molecules (Chen and Moe, 2015).

Among proposed mechanisms of pathological calcification in humans are deregulated calcium metabolism, chronic inflammation, smooth muscle cells reprogramming, and abnormal phosphate metabolism (Villa-Bellosta and Egido, 2017; Viegas et al., 2019). However, the comparison of tissue-specific mechanisms of calcification in humans remains largely understudied. To investigate these mechanisms we examined four tissues most commonly affected by pathological calcification in humans: artery aorta, artery tibial, pituitary and coronary artery. We found common deregulated pathways associated with inflammation and intracellular transport that can be therapeutically targeted.

## Materials and methods

### Subjects

We used publicly available data from the GTEx database.^1^ All GTEx tissue samples were derived by the GTEx consorcium from deceased donors, with age from 20 to 79, both men and women. GTEx get their study authorization *via* next-of-kin consent for the collection and banking of de-identified tissue samples for scientific research.

We analyzed histology slides and RNA-seq available through GTEx Portal for the following tissues: aortas, artery tibials, coronary arteries and pituitary glands. Detailed information about subjects, including sex, cause of death and age bracket can be found in the Supplementary Table 1, Pheno_data_GTEx.xls.

### Calcification grading

**Figure 1:**
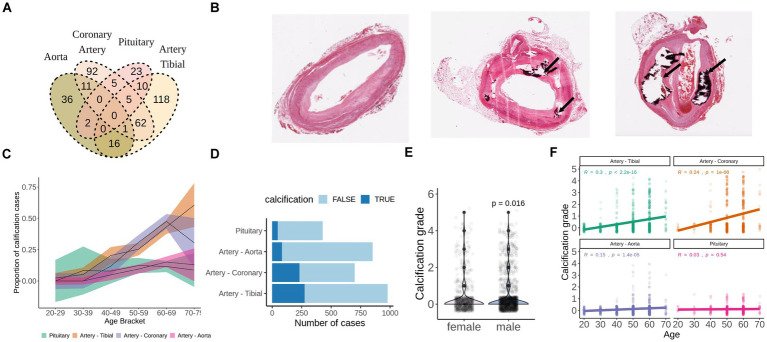
Calcification of soft tissues is a common age-related pathology. **(A)** Overlap of GTEx subjects who developed calcification in aorta, artery tibial, pituitary, or coronary artery as detected by H&E staining. **(B)** Representative examples of unaffected (grade 0), mildly calcified (grade 1-2) and severely calcified (grade 4) coronary arteries stained with H&E. Images were downloaded from GTEx Histology viewer. **(C)** Proportion of subjects with pathologically calcified tissues by age groups. Shadowed areas are 95% confidence intervals. **(D)** Calcification grade of men and women from GTEx database. *p*-value was calculated with wilcoxon test. **(E)** Calcification grade in aorta, artery tibial, coronary artery and pituitary by age group among GTEx subjects. **(F)** Total number of cases of pathological calcification by tissue. TRUE indicates presence of calcification, FALSE indicates absence of calcification.

H&E stainings of the aorta, artery tibial and coronary artery were accessed through GTEx Histology Viewer. Each sample was visually evaluated for the presence of calcification (dark-purple area or empty space surrounded by dark-purple area) and graded accordingly. Grade 1 corresponds to a small calcified area that occupies up to 5% of the section, grade 2 corresponds to a middle-sized calcified area that occupies up to 15% of the section, grade 3 corresponds to a large calcified area that occupies up to 25% of the section, grade 4 corresponds to the largest calcified area that occupies above 25% of the section (Figure 1B). Samples with non-vascular tissue contamination on H&E slides (mostly fat) were excluded due to possible contamination of fat tissue to RNA-seq data.

### Statistical methods

All statistical analysis was performed using the R language in Rstudio. We investigated linear relationships between transcript levels of protein-coding genes with calcification grade using sex, age bracket and cause of death as covariates using lm function in R. We further tested for pathway enrichment with gene set enrichment analysis (GSEA) using package clusterprofiler.

The Pearson correlation coefficients were calculated between expression of each gene and calcification grade. Statistical significance for relationship between calcification grade and sex as well as co-occurred pathological conditions were calculated with Mann–Whitney test due to non-normal distribution of calcification grades within compared groups.

### Gene expression analysis

After filtering, 430 aorta, 595 artery tibial, 124 coronary artery, and 283 pituitary samples were taken for gene expression analysis. RNA sequencing data was downloaded from the GTEx portal (GTEx_Analysis_2017-06-05_v8_RNASeQCv1.1.9_gene_reads.gct.gz). Gene counts were normalized and log-transformed using edgeR package in R, and were regressed versus calcification grade using sex, age bracket and cause of death as covariates in limma package. Analysis was restricted to protein-coding genes.

### GSEA

Rank for differentially expressed genes was calculated as -log10 (*p*-value) multiplied by 1 if logFC is positive, and multiplied by −1 if logFC is negative. Sorted gene lists with ranks were used for GSEA using clusterprofiler package in R.

## Results

### Vascular, but not pituitary, calcification is age-related pathology that primarily affects coronary and tibial arteries

We identified calcification deposits in 28 (6.5%) aortas, 121 (20%) artery tibials, 54 (43%) coronary arteries and 24 (8%) pituitary glands of GTEx subjects (Figure 1A). None of the subjects had calcification in all four tissues (Figure 1A), and subjects with calcification in the artery tibial were significantly more likely to have calcification in the coronary artery (OR = 2.56, *p* = 6.3e-07).

For all tissues, except for pituitary, the proportion of calcification cases and calcification grade correlated with age in concordance with the age-related nature of the pathology (Figures 1C,F). We also found that males had slightly higher calcification grade compared to females (*p* = 0.016) (Figure 1E). Calcification of artery tibial and coronary artery were the most prevalent in absolute numbers and in proportion (Figures 1C,D) and was present in over half of the GTEx subjects that are 60 years old or older (Figures 1C–E).

### Comparative analysis of differentially expressed genes and deregulated pathways in calcified soft tissues

**Figure 2:**
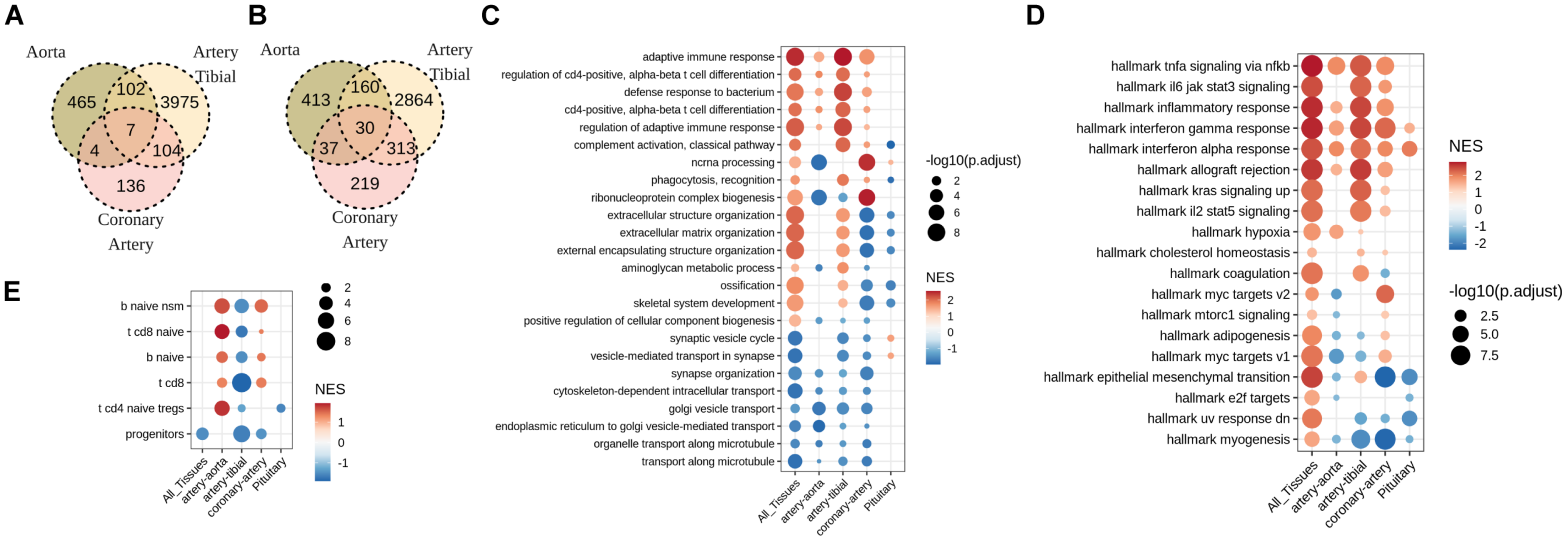
Soft tissues with pathological calcification up-regulate pathways involved in adaptive immune response, and down-regulate pathways of intracellular transport. **(A)** Overlap between up-regulated, and **(B)** down-regulated genes (FDR<0.05) in calcified aorta, artery tibial, and coronary artery. No statistically significant (FDR<0.05) gene changes were found in calcified pituitary. **(C)** GO and **(D)** MsigDB GSEA for differentially expressed genes in calcified aorta, artery tibial, coronary artery and pituitary, as well as all tissues combined together (All_Tissues) adjusted for sex, age, and HardyScale of GTEx subjects. **(E)** GSEA for custom-created gene markers of human immune cell populations. Size of the dot is -log10 of FDR-adjusted p-values, and color is normalized enrichment score.

To establish differentially affected genes in calcified tissues, expression of each gene was associated with calcification grade using sex, age, and cause of death as a covariate. None of the genes were significantly associated (FDR < 0.05) with calcification grade in pituitary glands. Unexpectedly, DEGs associated with calcification minimally overlap across vessels. Only 7 genes were commonly up-regulated (Figure 2A) and 30 down-regulated (Figure 2B) in calcified artery tibial, coronary artery and aorta.

Despite low overlap of individual DEGs across tissues, GSEA revealed common pathways that were deregulated across tissues. Among these, down-regulated pathways were related to cell transport while up-regulated pathways included immune response, inflammation, in particular regulation of leukocyte activation and T-cell differentiation (Figures 2C,D). One prominent difference between tibial and coronary artery was deregulation of extracellular matrix, where calcified tibial up-regulated and coronary artery down-regulated genes involved in this pathway (Figures 2C,D). To follow up on inflammatory signature of calcified tissues, we tested enrichment of DEGs observed with calcification in each tissue in signatures of immune cell types. Interestingly, we observe significant enrichment in T and B cell signatures, and not myeloid signatures (Figure 2E). Furthermore, signs of enrichment differed across tissues, where calcified tibial arteries were negatively enriched in T and B-cell related signatures, while calcified coronary arteries and aorta were positively enriched in both (Figure 2E).

### Genes involved in ossification and T-cell mediated immune response are deregulated in calcification in tissue-specific manner

**Figure 3:**
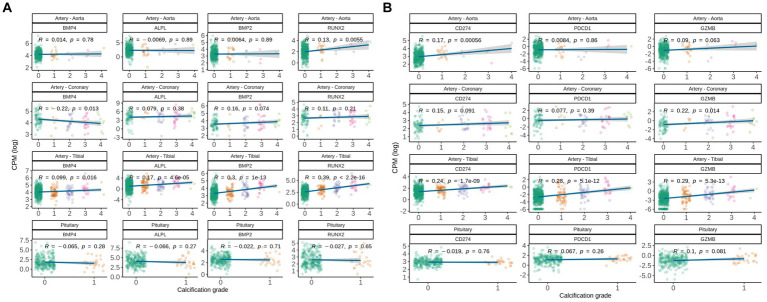
Gene expression correlation between manually assigned calcification grade in human soft tissues for subjects from GTEx database and **(A)** expression levels of genes known to be involved in pathological and natural calcification, and **(B)** expression levels of gene associated with immune-associated inflammation. Pearson correlation coefficients are shown.

We further investigated expression levels of genes involved in the healthy ossification process (*BMP2, BMP4, RUNX2, ALPL*) and T-cell mediated immune response (*GZMB*, *CD274*, *PDCD1*). Interestingly, classical regulators of ossification in the bone were up-regulated in artery tibial and aorta, but not in coronary artery and pituitary (Figure 3A), therefore we can speculate that soft tissues might engage into different paths of pathological ossification.

Following up on commonly deregulated T-cell signatures in calcified tissues, we analyzed *CD274, PDCD1*, and *GZMB*. Interestingly, *CD274* (encodes PD-L1) was up-regulated with calcification grade in artery tibial and aorta, but not in coronary artery and pituitary (Figure 3B). Calcified artery tibial further up-regulated *PDCD1* (encodes PD-1) and *GZMB*, suggesting that adaptive immune cells are involved in pathologic calcification.

To differentiate local and global deregulation of adaptive immune response we analyzed gene expression changes in the blood of the same subjects. We correlated expression of each gene in the blood with calcification grade in artery tibial and aorta and used sex, age and cause of death as a covariate. None of the genes in blood were significantly associated (FDR < 0.05) with calcification grade and none of the pathways were significantly enriched (data not shown). This indicates that deregulation of adaptive immune response observed in calcified vessels happens locally in the soft tissue rather than globally.

### Atherosclerosis is co-occurring with calcification across all three blood vessels

**Figure 4:**
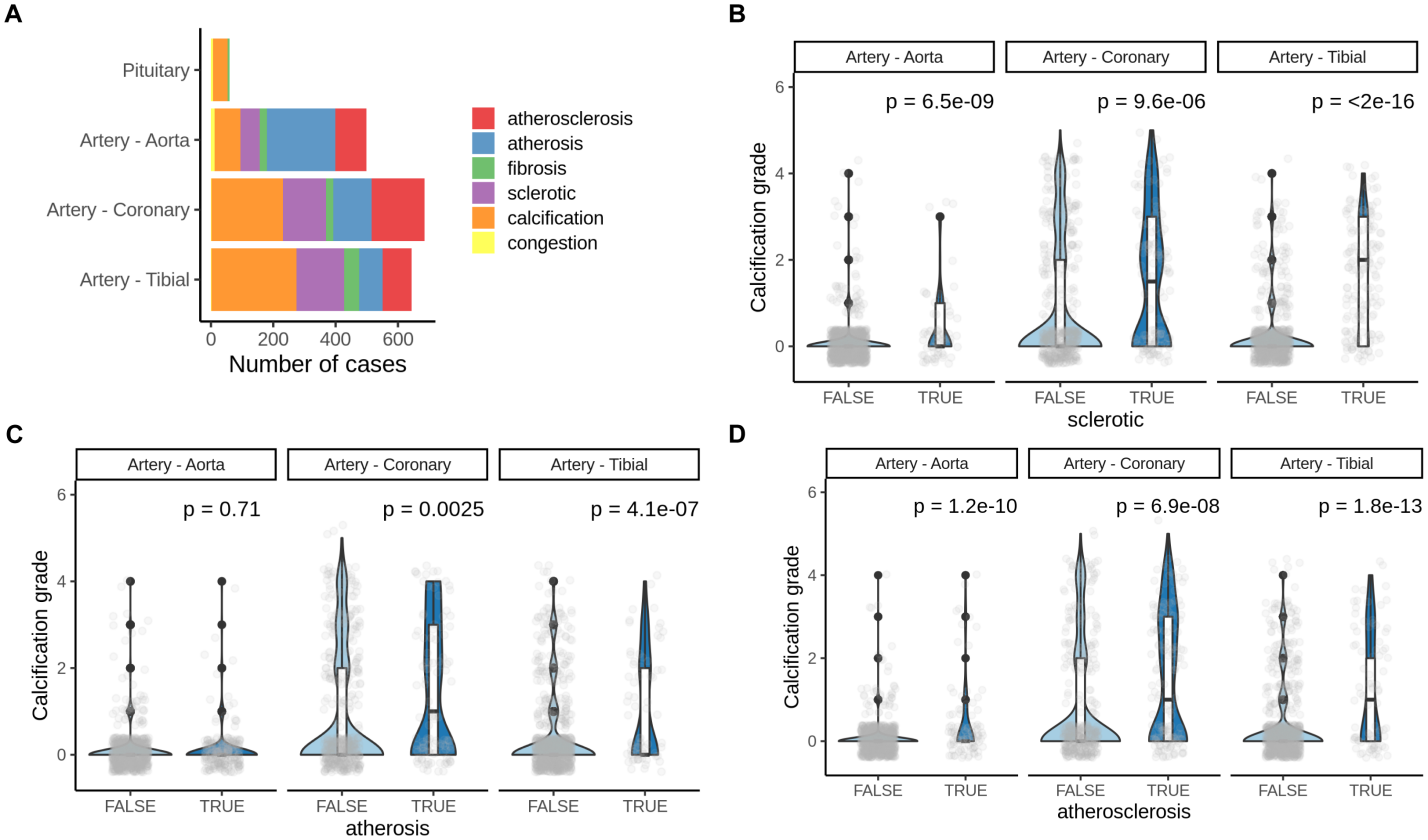
Pathologies co-occurring with pathological calcification. **(A)** Number of most common pathologies detected by H&E staining of corresponding human tissues. **(B)** Calcification grade in aorta, coronary artery and artery tibial that also presents **(B)** sclerotic changes, **(C)** arthrosis or **(D)** atherosclerosis. TRUE–indicate the presence of corresponding pathogenic change and, FALSE–indicates the absence of it. *p*-value was calculated using the Mann–Whitney test.

We further analyzed pathologies that co-occur with calcification in all four soft tissues. Atherosclerosis, arthrosis, and sclerotic lesions were commonly detected by pathologists in H&E slides in all three vessels but not in pituitary (Figure 4A).

We found significant increase in calcification grade with presence of sclerotic, arthrosis lessons, and with atherosclerosis in all three tissues (artery tibial, artery coronary, artery aorta’ Figures 4B,D), except for arthrosis in aorta (Figure 4C). That suggests that atherosclerotic changes in vessels usually co-occur with vascular calcification and might share mechanisms of development.

## Conclusion

Here we performed the first comparative analysis of gene expression and pathological changes that accompany calcification of coronary and tibial artery, aorta and pituitary in human subjects. We confirmed an increase in prevalence and grade of vascular calcification with age, as previously reported (Tesauro et al., 2017; Pescatore et al., 2019). Subjects with calcification in the artery tibial were more likely to have calcification in the coronary artery, but not in the aorta. Gene expression changes associated with calcification in tibial and coronary arteries did not reveal any specific pathways that would differentiate them from calcification in aorta. This indicates that calcified lesions in the tibial and coronary artery may share risk factors rather than specific molecular mechanisms.

Calcified aorta, tibial and coronary arteries poorly overlapped by DEGs, however, largely overlapped by pathways, and demonstrated deregulated adaptive immune response and engagement in classical ossification pathways as possible drivers in these tissues. Calcified aorta stood out and shared little DEGs with other soft tissues, emphasizing different molecular etiology and that patients with vascular calcification could benefit more from personalized tissue-specific treatments. These results are supported by previous findings (Sawabe, 2010; Lee et al., 2013).

We observed upregulation of BMP2 in calcified artery tibial in comparison with healthy control, which is in line with recent research (Nakagawa et al., 2010; Derwall et al., 2012; Kwon et al., 2017).

*RUNX2* is a well described transcriptional regulator that causes vascular calcification in mice (Sun et al., 2012; Lin et al., 2015). The levels of RUNX2 were significantly upregulated in the tibial aorta and in the artery aorta of humans, but not in the coronary artery, indicating that mechanisms of pathological ossification differ across vascular tissues.

MGP, a well-known inhibitor of calcification, is a protein that is secreted by various cell types and dependent on vitamin K. It can be found in VSMCs, macrophages, and osteoblasts, as well as all tissues (Ngai et al., 2018; Bjørklund et al., 2020).

It was observed that in healthy vessels, there is a gradient where MGP levels are highest on the innermost side and fall toward the middle layer. MGP is typically expressed by VSMCs and the fibrous cap of atherosclerotic lesions. According to Engelse et al. (2001) there is a negative link between zones of decreased MGP expression and increased calcification in the inner and middle layers of the vessel, as well as Runx2.

Jaminon et al. (2020) showed that the levels of dp-ucMGP in plasma are a reliable, independent indicator of increased VC in patients with CKD5 (chronic kidney disease). They also have a strong correlation with higher CAC scores and the extent of medial calcification. Furthermore, a high expression of MGP in the vascular system is linked to higher CAC scores and plasma dp-ucMGP levels.

Surprisingly, MGP was significantly upregulated in calcified artery tibial, but downregulated in calcified coronary artery, which is in line with previous systematic review underlying conflicting results across tissues (Barrett et al., 2018).

In the work of Jono et al. (2004) it was found that serum MGP negatively correlates with CAC score. The coronary artery calcium (CAC) is a highly accurate indicator of coronary artery malfunction. Through various studies it has been established that CAC scoring is a reliable and effective method of assessing the risk of major cardiac events, particularly in asymptomatic individuals who are planning primary prevention interventions. Additionally, CAC testing is cost-effective for asymptomatic groups with varying levels of baseline risk (Shreya et al., 2021).

In patients with coronary artery calcification was lower than in the control group, which indicates that MGP is not a universal marker or regulator of vascular calcification and might be involved in coronary artery calcification rather than in artery tibial one.

Gene expression changes associated with calcification across blood vessels and pituitary overlapped by enriched Kyoto Encyclopedia of Genes and Genomes (KEGG) and GO pathways Down-regulated pathways were cell adhesion pathways while up-regulated pathways included inflammation, especially involved in regulation of leukocyte activation and T-cell differentiation.

Finally, visceral adiposity and insulin resistance frequently coexist in patients with VC (Shang et al., 2016), as well as atherosclerosis (Doherty et al., 2003). Surprisingly, vascular calcification was the most prevalent among pathologies found in the artery tibial of GTEx subjects. It raises the possibility that some vascular calcification cases develop independently of atherosclerosis or arthrosis. One explanation is that the artery tibial is more susceptible to Mönckeberg sclerosis that develops in the media layer of artery and thus have independent etiology from the intima-associated atherosclerosis. On the other hand, our analysis is based on pathology notes which aren’t protected from human errors. Future studies may develop automatic algorithms to detect pathologies from H&E stainings to validate human annotations and our findings.

We also found a positive association between gene expression of *PDCD1* (PD-L1) and *CD274* (PD-1) with calcification grade in artery tibial, and up-regulation of *CD274* in aorta. Furthermore, GSEA analysis of genes deregulated with calcification were consistently revealing association between vascular calcification and adaptive immune response, but not innate immune response. This finding is unexpected since vascular pathologies are often discussed in the context of infiltration of macrophages rather than T or B cells. Previous analysis of calcified aortic valves revealed T-cell signature (Guauque-Olarte et al., 2016), which is consistent with our finding in aorta and coronary arteries. Furthermore, increased levels of auto (Erkhem-Ochir et al., 2020) antibodies generated by B cells were previously found in subjects with coronary artery calcification (Geraldino-Pardilla et al., 2017), in line with our observations of B cell signature found in coronary artery calcification, and also in aorta.

Furthermore, PD-L1 protein level was previously shown to be positively associated with aortic valve calcification (Erkhem-Ochir et al., 2020). Here we show that *CD274* (encodes PD-L1) was up-regulated with calcification grade in artery tibial and aorta, but not in coronary artery and pituitary. According to Okazaki and Honjo (2007), Gotsman et al. (2007), Weyand et al. (2018), and Sun et al. (2020), Bu et al. (2011) alterations in PD-1 or PD-L1 expression can influence the degree of inflammation and condition of coronary plaques in atherosclerosis. Granzyme B (*GZMB*) is a protease secreted by T cells and NK cells and is known for its pro-apoptotic properties (Voskoboinik et al., 2015; Guo et al., 2022). Previous by qPCR and immunohistochemistry data confirmed that *GZMB* expression was higher in calcified aortic valve tissues than in control valves (Ohukainen et al., 2015; Guo et al., 2022), which is in line with our results, where we found correlations between *GZMB* expression and calcification grade in coronary and tibial artery. Furthermore, we have not found signs of adaptive immune response deregulation in the blood of the subject with vascular calcification. This indicates that the adaptive immune system locally contributes to pathogenesis of vascular calcification. Future studies might focus on the importance of local adaptive immune response in pathogenesis of vascular calcification and dissect bulk gene expression signature by performing single-cell RNA sequencing of calcified vessels.

## Data availability statement

Publicly available datasets were analyzed in this study. This data can be found here: https://gtexportal.org/home/.

## Author contributions

AI and AS contributed to conception and design of the study and wrote the first draft of the manuscript. AI organized the database. AS performed the statistical analysis. AI, AS, AP, AZ, YS, ND, and AK wrote sections of the manuscript. All authors contributed to manuscript revision, read, and approved the submitted version.

## Conflict of interest

AI and AP are employed by MineGenics LLC. AS is employed by Retro Biosciences Inc.

The remaining authors declare that the research was conducted in the absence of any commercial or financial relationships that could be construed as a potential conflict of interest.

## Publisher’s note

All claims expressed in this article are solely those of the authors and do not necessarily represent those of their affiliated organizations, or those of the publisher, the editors and the reviewers. Any product that may be evaluated in this article, or claim that may be made by its manufacturer, is not guaranteed or endorsed by the publisher.

## Footnotes

^1^https://gtexportal.org/home/aboutGTEx
