# Supplementary material for: Comparative analysis of calcified soft tissues revealed shared deregulated pathways
Source: Front Aging Neurosci. 2023 Jun 14;15:1131548. doi: 10.3389/fnagi.2023.1131548 (PMC10335799; doi:10.3389/fnagi.2023.1131548)
Supplement: Supplementary file 2 [file Presentation_1.PPTX]

## Slide 1
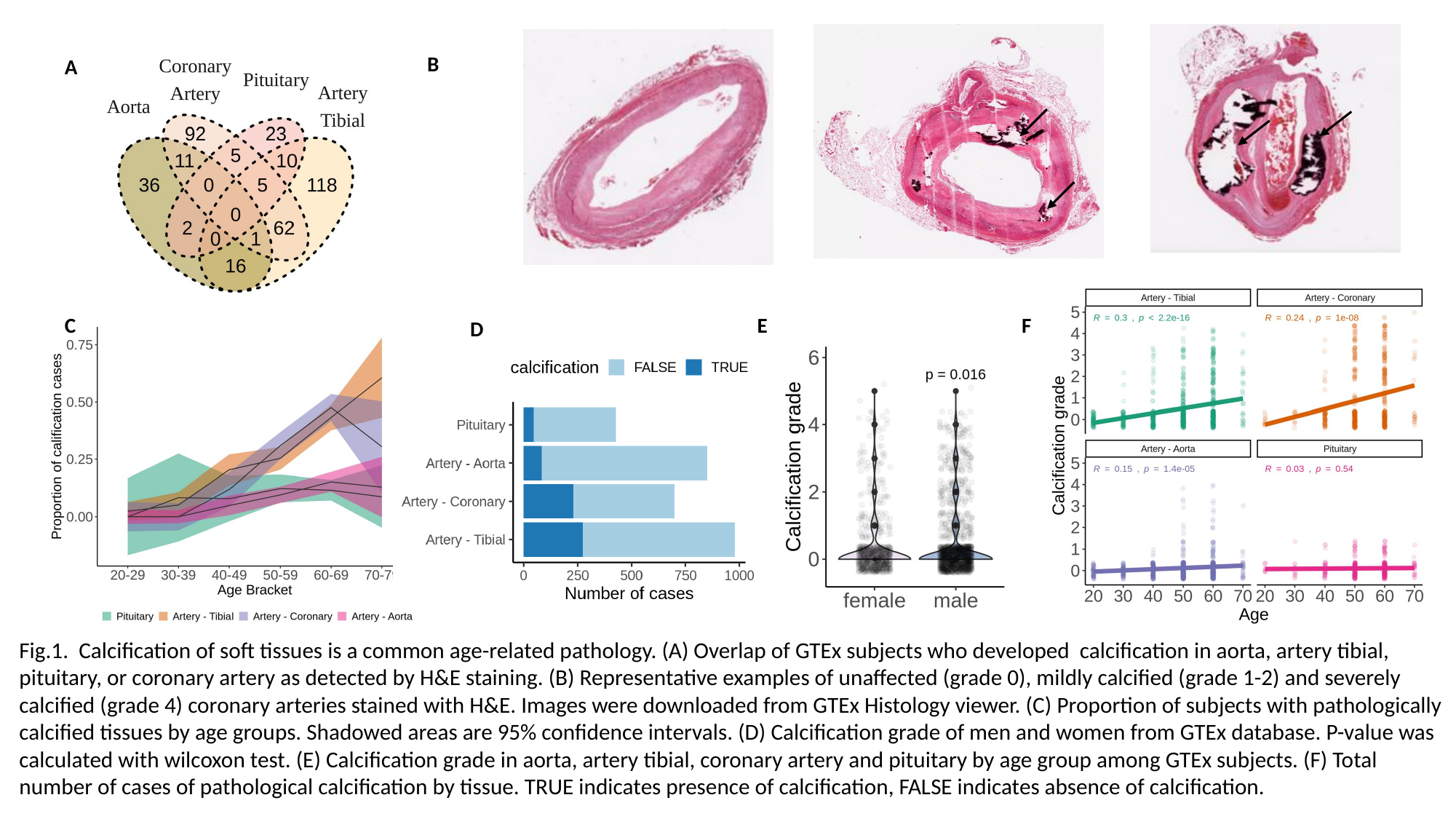

B
A
C
E
F
D
Fig.1. Calcification of soft tissues is a common age-related pathology. (A) Overlap of GTEx subjects who developed calcification in aorta, artery tibial, pituitary, or coronary artery as detected by H&E staining. (B) Representative examples of unaffected (grade 0), mildly calcified (grade 1-2) and severely calcified (grade 4) coronary arteries stained with H&E. Images were downloaded from GTEx Histology viewer. (C) Proportion of subjects with pathologically calcified tissues by age groups. Shadowed areas are 95% confidence intervals. (D) Calcification grade of men and women from GTEx database. P-value was calculated with wilcoxon test. (E) Calcification grade in aorta, artery tibial, coronary artery and pituitary by age group among GTEx subjects. (F) Total number of cases of pathological calcification by tissue. TRUE indicates presence of calcification, FALSE indicates absence of calcification.

## Slide 2
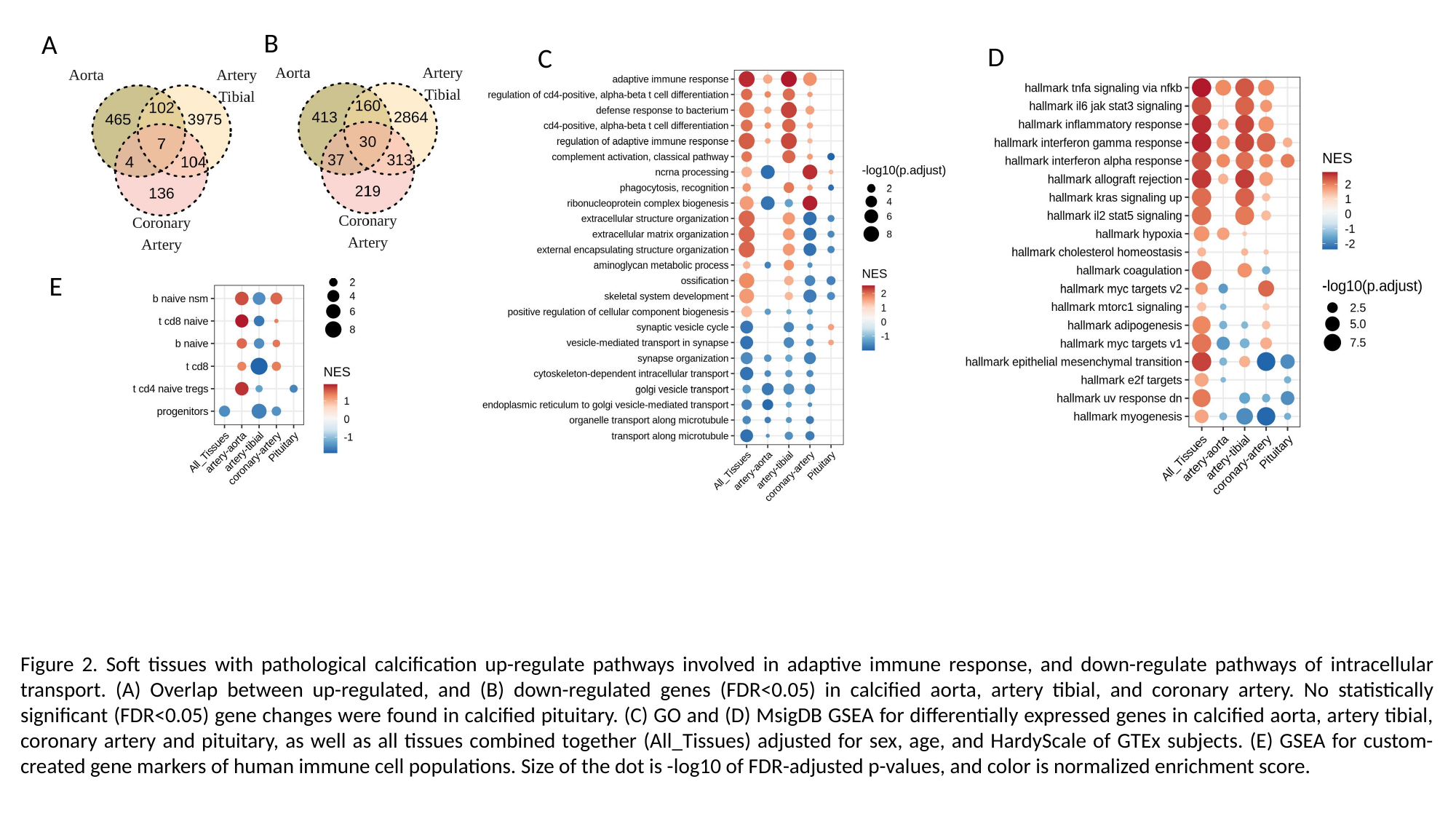

B
A
D
C
E
Figure 2. Soft tissues with pathological calcification up-regulate pathways involved in adaptive immune response, and down-regulate pathways of intracellular transport. (A) Overlap between up-regulated, and (B) down-regulated genes (FDR<0.05) in calcified aorta, artery tibial, and coronary artery. No statistically significant (FDR<0.05) gene changes were found in calcified pituitary. (C) GO and (D) MsigDB GSEA for differentially expressed genes in calcified aorta, artery tibial, coronary artery and pituitary, as well as all tissues combined together (All_Tissues) adjusted for sex, age, and HardyScale of GTEx subjects. (E) GSEA for custom-created gene markers of human immune cell populations. Size of the dot is -log10 of FDR-adjusted p-values, and color is normalized enrichment score.

## Slide 3
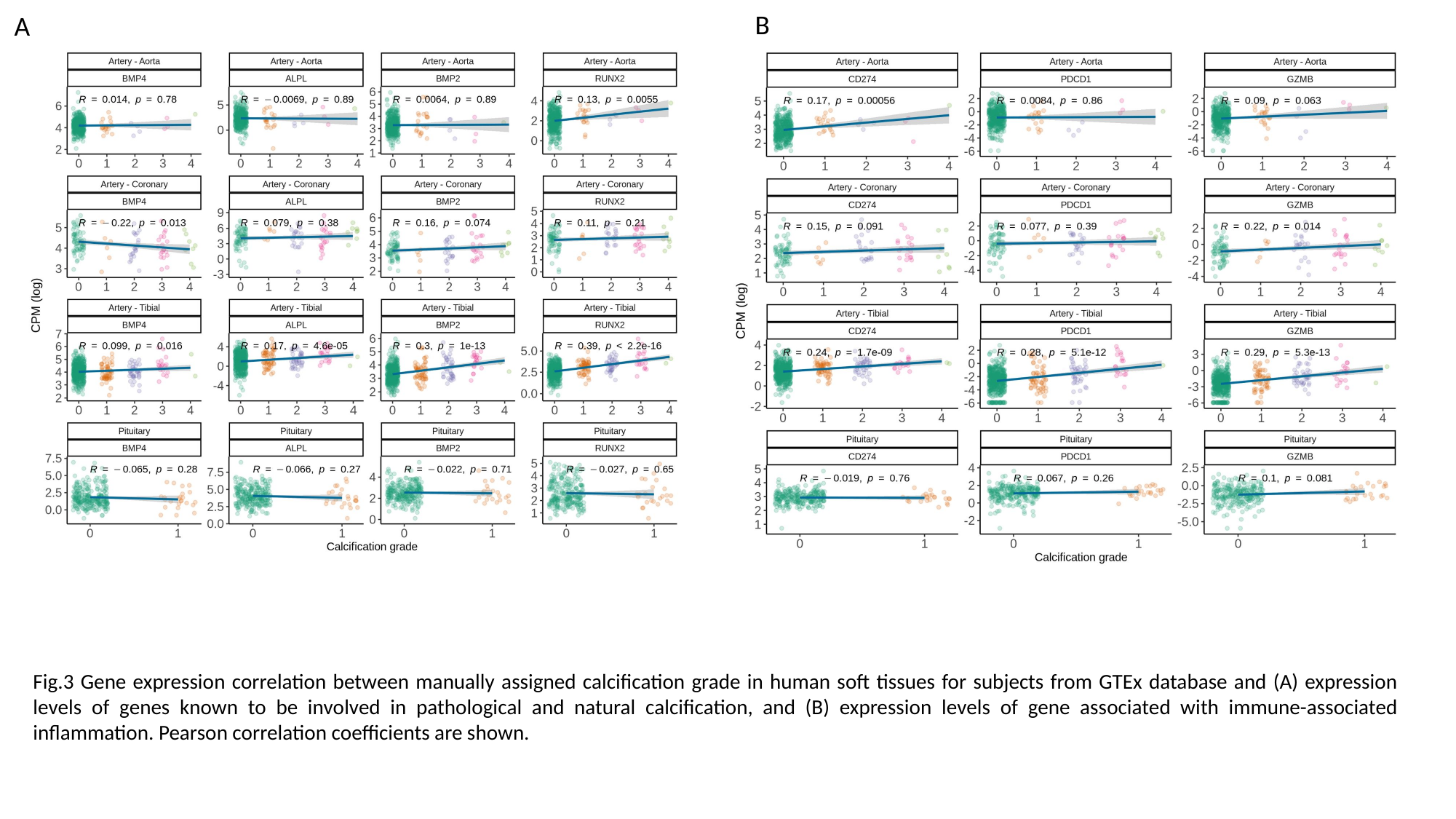

B
A
Fig.3 Gene expression correlation between manually assigned calcification grade in human soft tissues for subjects from GTEx database and (A) expression levels of genes known to be involved in pathological and natural calcification, and (B) expression levels of gene associated with immune-associated inflammation. Pearson correlation coefficients are shown.

## Slide 4
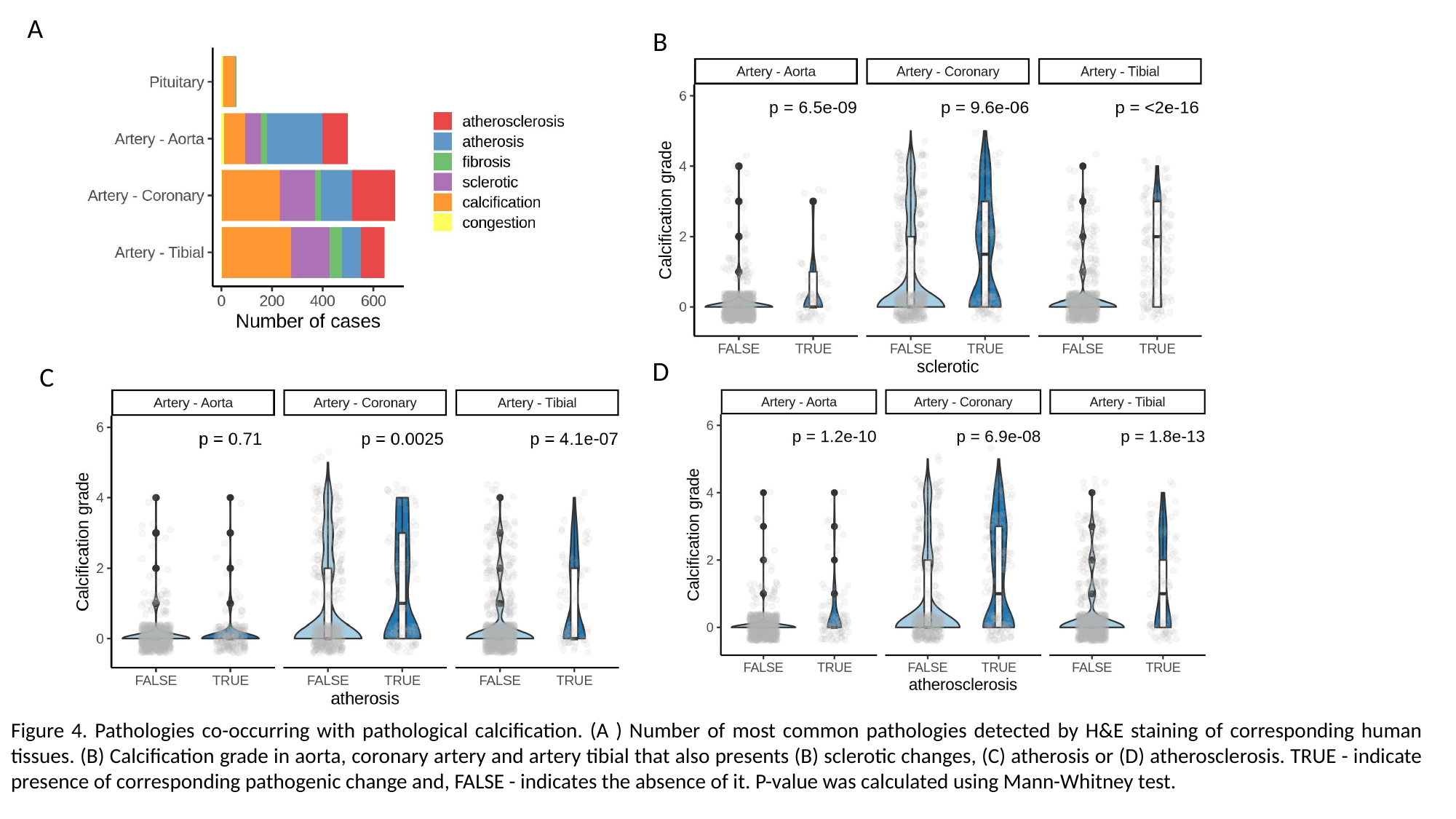

A
B
D
C
Figure 4. Pathologies co-occurring with pathological calcification. (A ) Number of most common pathologies detected by H&E staining of corresponding human tissues. (B) Calcification grade in aorta, coronary artery and artery tibial that also presents (B) sclerotic changes, (C) atherosis or (D) atherosclerosis. TRUE - indicate presence of corresponding pathogenic change and, FALSE - indicates the absence of it. P-value was calculated using Mann-Whitney test.
